# ^177^Lutetium SPECT/CT: Evaluation of collimator, photopeak and scatter correction

**DOI:** 10.1002/acm2.12991

**Published:** 2020-08-13

**Authors:** Daphne M. V. Huizing, Michiel Sinaasappel, Marien C. Dekker, Marcel P. M. Stokkel, Berlinda J. de Wit – van der Veen

**Affiliations:** ^1^ Department of Nuclear Medicine Netherlands Cancer Institute Amsterdam The Netherlands; ^2^ Department of Physics Netherlands Cancer Institute Amsterdam The Netherlands

**Keywords:** ^177^Lutetium, quantitative imaging, radionuclide therapy, SPECT/CT

## Abstract

**Purpose:**

The goal of this study was to find the optimal combination of collimator, photopeak and scatter correction for ^177^Lutetium (^177^Lu) SPECT/CT imaging.

**Methods:**

Three experiments [sphere‐to‐background ratios (SBR) 50:1, 10:1, and 2:1] were performed with the NEMA Image Quality phantom filled with ^177^Lu‐trichloride. SPECT/CT acquisitions were performed with the medium‐energy low‐penetration (MELP) collimator and ^99m^Tc/Krypton collimator. For each acquisition six reconstructions, all with attenuation correction (AC), were made: the 113‐keV photopeak only, the 208‐keV photopeak only and both photopeaks combined, each with or without scatter correction (SC). Image quality was assessed using contrast‐to‐noise ratios (CNR), quantification accuracy by means of recovery coefficients (RCs) and the spatial resolution using line profiles.

**Results:**

With SBR 50:1 and 10:1, both collimators met the *Rose criterion* (CNR > 5), whereas the MELP collimator showed a higher CNR for the 2:1 ratio. The RC_mean_ was higher with the MELP collimator, most explicit after the 208‐keV AC/SC reconstruction for all acquisitions. The line profiles showed a better spatial resolution for the MELP collimator and the 208‐keV AC/SC reconstructions.

**Conclusion:**

^177^Lu SPECT/CT image quality and quantification was most optimal when acquired with the MELP collimator and reconstructed using the 208‐keV photopeak, with AC and SC.

**Abbreviations**
AC: attenuation correction
AC/SC: attenuation and scatter correction
CF: calibration factor
CNR: contrast‐to‐noise ratio
CT: computed tomography
EANM: European Association of Nuclear Medicine
EARL: EANM Research Ltd
^177^Lu: ^177^Lutetium
MELP: medium energy low penetration
MIRD: Medical Internal Radiation Dose
PVE: partial volume effect
RC: recovery coefficient
RNT: radionuclide therapy
SBR: sphere‐to‐background ratios
SC: scatter correction
SPECT: single positron emission computed tomography
VOI: volume‐of‐interest

## 1 INTRODUCTION

In recent years, radionuclide therapy (RNT) boosted the field of nuclear medicine with ^177^Lutetium (^177^Lu)‐DOTATATE for patients with neuroendocrine tumors.^1^ In the upcoming years, treatment with ^177^Lu‐labeled prostate specific membrane antigen for patients with metastatic prostate cancer is likely to further increase RNT.^2^ In this respect, most centers use a “one‐size‐fits‐all” approach for the administered amount of radioactivity during RNT, although treatment response between patients varies and patients might be undertreated due to this approach.^3^ Dosimetry, which refers to the assessment of the absorbed dose in tissues, could aid in personalized RNT by increasing the dose to the tumor while minimizing irradiation of organs at risk.^4^

Quantitative SPECT/CT imaging is important for ^177^Lu dosimetry and although numerous articles are already available, a clear description for clinical practice is lacking and many sites use different methods.^5^, ^6^, ^7^, ^8^, ^9^, ^10^ Acquisition and reconstruction protocols are roughly based on collimator, photopeak definition, and corrections. The medium‐energy low‐penetration (MELP) collimator is advised for ^177^Lu gamma imaging because of its lower septal penetration.^11^ In our institute, a particular low‐energy high‐resolution collimator with thick septa (^99m^Tc/Krypton) is available, with similar specifications compared to the MELP collimator (Table 1). ^177^Lu has two main photopeaks at 113 keV (6.2%) and 208 keV (10.4%).^7^ According to the MIRD/EANM guidelines, the 208‐keV photopeak is preferable for imaging with MELP collimators and the 113‐keV photopeak for LEHR collimators.^7^ Combining the counts from both photopeaks boosts the overall signal, which could be beneficial in late imaging time points.^12^ Next to the routinely used attenuation correction (AC) for SPECT/CT imaging, also scatter correction (SC) is suggested to improve the ^177^Lu quantification.^13^ As the ^177^Lu photopeaks have quite different energies, multiple scatter windows are applied to correct for this image degrading effect.

**Table 1:** Specifications of the low energy high resolution (^99m^Tc/Krypton) and medium energy low penetration (MELP) collimator.

Specification	^99m^Tc/Krypton	MELP
Hole size (mm)	2.5	2.95
Septal thickness (mm)	0.4	1.14
Hole length (mm)	40	40.6
210 keV		
System resolution (FWHM at 10 cm)	10.0	12.3
System sensitivity (cts/min/MBq)	17.3	16.3
Septal penetration (%)	5.6	0.14
140 keV		
System resolution (FWHM at 10 cm)	10.0	12.3
System sensitivity (cts/min/MBq)	17.3	16.3
Septal penetration (%)	0.02	<0.01
Weight (kg)	45	63.5

Aside from absolute quantification of uptake, visual assessment of accumulation in tissues and delineation of lesions is important in clinical practice. Physicians are used to visually assess normal tissue to tumor ratios, where uptake in small tumors is hampered by image contrast, noise and spatial resolution. So, it is important to recognize that a compromise between these factors has to be made when selecting the optimal imaging and reconstruction parameters for ^177^Lu dosimetry.

The goal of this study was to find the optimal combination of collimator, photopeak, and scatter correction for ^177^Lu SPECT/CT imaging. Different protocols were compared with respect to contrast‐to‐noise ratios (CNR), quantification accuracy by means of recovery coefficients (RCs), and spatial resolution using line profiles.

## 2 MATERIALS AND METHODS

### 2.A Phantom image acquisition and reconstruction

Experiments were performed using the NEMA NU2‐2012 Image Quality phantom (PI Medical Diagnostic Equipment B.V., Raamsdonksveer, The Netherlands). The specific activity concentrations used in this study were locally acquired from a research ^177^Lu‐trichloride batch vial (IDB Holland, Baarle‐Nassau, The Netherlands). Spheres of the phantom (sizes: 13, 17, 22, 28, and 37 mm diameter) were filled with ~1.0 MBq/ml and activity was added to the background compartment to obtain three different sphere‐to‐background ratios (SBR) of 50:1, 10:1, and 2:1. These ratios were chosen as they roughly concur with clinical accumulation in tumors compared to the blood pool, kidney, and liver.

All acquisitions were performed on a Symbia T SPECT/CT system (Siemens, Erlangen, Germany) with two collimator types: MELP and ^99m^Tc‐Krypton (specifications are shown in Table 1). Acquisition settings included continuous mode, 13 s/view, and 48 views/head using a noncircular orbit and 180° detector configuration (128 × 128 matrix). Primary energy windows included the 113 keV ± 10% photopeak (101.7–124.3 keV) and 208 keV ± 10% photopeak (187.2–228.8 keV). Two scatter windows were additionally defined: a 10% downscatter window below the 208‐keV photopeak (166.4–187.2 keV) and a general scatter window 50 keV ± 50% (25.0–75.0 keV). This method of scatter correction is described in the paper of Zeintl et al. in more detail.^14^ All SPECT reconstructions were performed using 3D‐OSEM (FLASH 3D) with 10 iterations and 8 subsets, with an 8.4‐mm Gaussian filter, and resulted in cubic 4.8 × 4.8 × 4.8 mm voxels. For each acquisition six reconstructions, all with AC, were made: the 113‐keV photopeak only, the 208‐keV photopeak only, and both photopeaks combined, each with or without SC. Low‐dose CT images for AC were acquired with 130 kV and 40 mAs.

### 2.B Imaging analysis

Calibration factors (CFs) to convert counts to activity concentration were determined using multiple large volumes‐of‐interest (VOIs) of ~100 ml, randomly placed in the background of the phantom. Calibration factors were calculated for each specific combination of collimator, photopeak, and the presence of scatter correction, in concordance with the MIRD pamphlet no. 23 recommendations,^9^ see Eq. (1):

(1) $$CF=\frac{C}{A}$$

with C the average number of counts in a certain volume with known activity concentration A. Image quality was assessed using CNR calculated according to Eq. (2):

(2) $$CNR=\frac{C_{S}‐C_{B}}{\sigma_{B}}$$

where $C_{S}$ represents the average number of counts in each sphere, $C_{B}$ the average number of counts in the background volumes used to determine the CF, and $\sigma_{B}$ the average standard deviation of the background volumes.^15^ The *Rose criterion*, CNR > 5, was used to classify whether an object is detectable or not.^16^ Absolute quantification was evaluated using the average recovery coefficient (RC_mean_) according to Eq. (3)9:

(3) $$RC_{\mathrm{mean}}=\frac{S_{\mathrm{estimated}}}{S_{\mathrm{true}}}$$

with $S_{\mathrm{estimated}}$ the average measured activity concentration (kBq/ml) in the sphere and $S_{\mathrm{true}}$ the known activity concentration. The volume of the sphere and the according average number of counts in the sphere was determined based on CT dimensions. Lastly, line profiles were drawn through the 37 and 17 mm sphere to visualize spatial resolution at the transition between sphere and background.

## 3 RESULTS

**Fig. 1:**
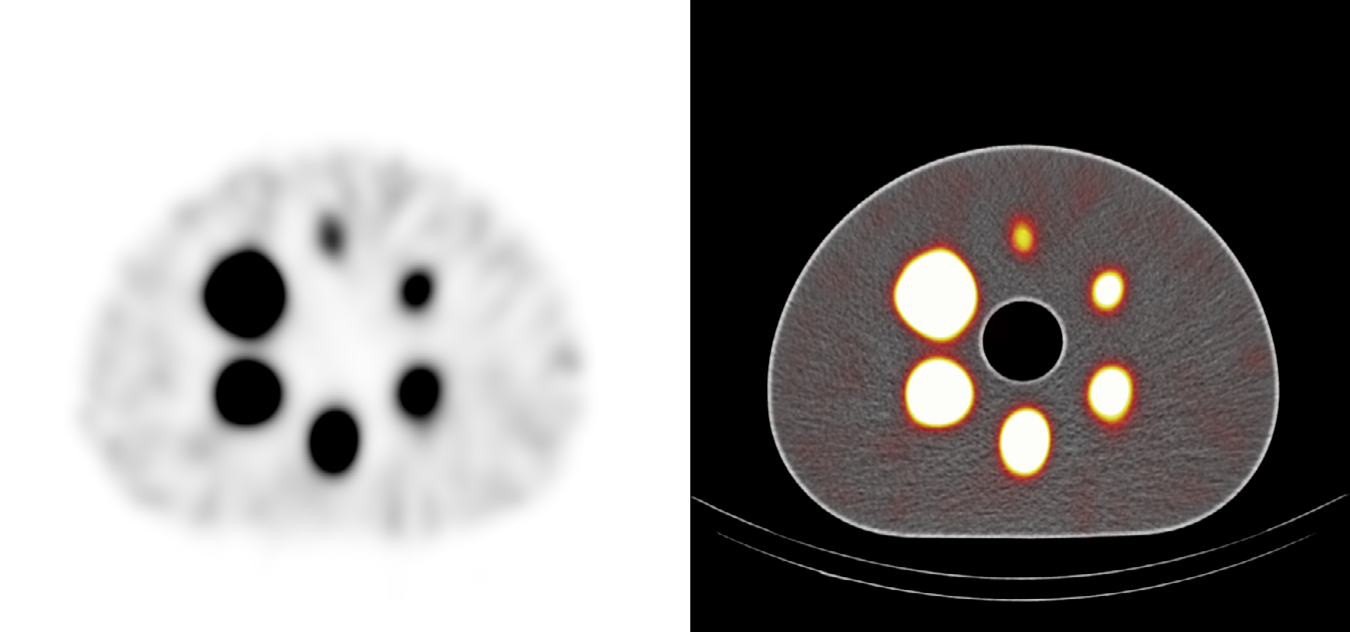
Example of the NEMA image quality phantom (medium energy low penetration collimator, 208 keV AC/SC).

**Fig. 2:**
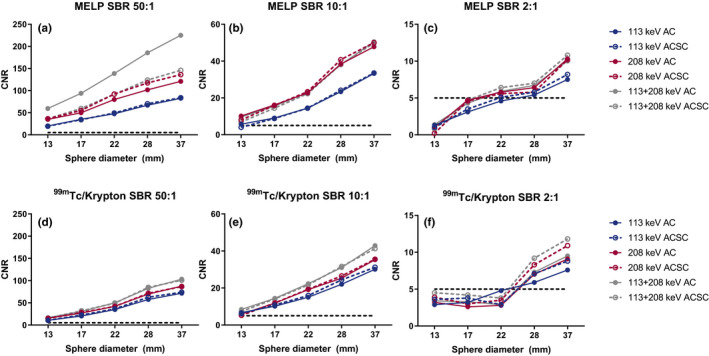
Contrast‐to‐noise over all spheres and reconstructions based on the average sphere and background activity concentration. The dotted line represents contrast‐to‐noise ratios (CNR) = 5 according to the *Rose criterion*.^14^ The highest CNR can be observed from the medium energy low penetration collimator and the 113 + 208 keV attenuation correction/scatter correction reconstruction.

An example of the NEMA Image Quality phantom, acquired with the MELP collimator and 208 keV with attenuation correction and scatter correction (AC/SC), is shown in Fig. 1. The CNR for all acquisitions are shown in Fig. 2, whereas Table 2 provides the CNR values of both collimators with all reconstructions for the 37 mm sphere. All reconstructions of SBR 50:1 and 10:1 comply with the *Rose criterion* for both collimators. For SBR 2:1, however, only spheres ≥ 22 mm and spheres ≥ 28 mm comply for the MELP collimator and ^99m^Tc/Krypton collimator, respectively.

**Table 2:** Contrast‐to‐noise ratios of the 37 mm sphere.

Collimator	MELP			^99m^Tc‐Krypton		
Reconstruction	SBR 50:1	SBR 10:1	SBR 2:1	SBR 50:1	SBR 10:1	SBR 2:1
113 AC	82.7	33.3	7.5	71.6	30.1	7.6
113 AC/SC	83.8	33.5	8.2	73.8	31.2	8.8
208 AC	121.0	47.8	10.3	87.5	35.3	9.1
208 AC/SC	136.2	50.0	10.2	86.6	35.5	10.9
113 + 208 AC	225.0	50.5	10.0	103.3	42.9	9.5
113 + 208 AC/SC	145.9	49.8	10.8	101.0	41.3	11.8

**Fig. 3:**
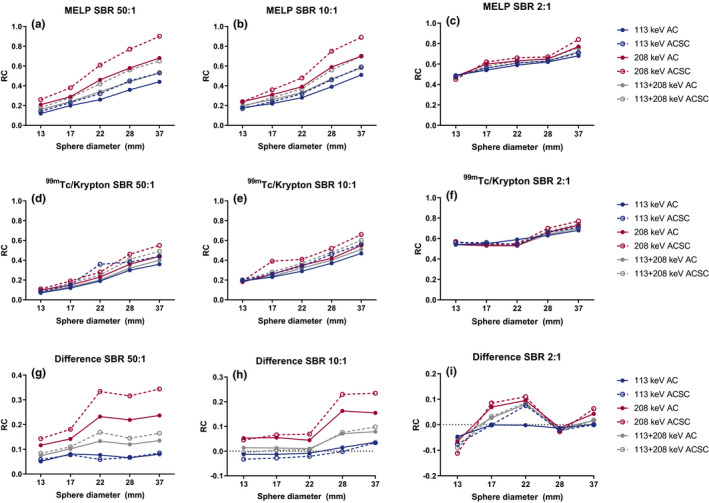
RC_mean_‐curves based on the average activity concentrations of the medium energy low penetration (MELP) (a–c) and ^99m^Tc‐Krypton (d–f) collimator. Figure (g)–(i) show the difference between both collimators. The MELP collimator shows the highest recovery, as well as the 208 keV attenuation correction/scatter correction reconstruction.

All RC_mean_ curves are shown in Fig. 3 and the absolute values for the 37‐mm sphere are provided in Table 3. Figures 2(g)–(I) show the difference between both collimators, where a positive value indicates a higher recovery for the MELP collimator. Overall, the RC_mean_ of the MELP collimator was higher compared to the ^99m^Tc/Krypton collimator. The 208‐keV AC/SC reconstruction showed the highest recovery for all SBR. All AC/SC reconstructions demonstrated higher recoveries compared to the solely AC reconstructed images. All reconstructions for SBR 50:1 and most reconstructions for SBR 10:1 are in favor for the MELP collimator, whereas the recovery of the 13‐mm sphere with SBR 2:1 is higher for the ^99m^Tc‐Krypton collimator.

**Table 3:** Recovery coefficients of the 37 mm sphere

Collimator	MELP			^99m^Tc‐Krypton		
Reconstruction	SBR 50:1	SBR 10:1	SBR 2:1	SBR 50:1	SBR 10:1	SBR 2:1
113 AC	0.44	0.51	0.68	0.36	0.47	0.68
113 AC/SC	0.53	0.59	0.71	0.44	0.56	0.71
208 AC	0.68	0.70	0.77	0.44	0.55	0.73
208 AC/SC	0.90	0.89	0.84	0.55	0.66	0.77
113 + 208 AC	0.53	0.58	0.72	0.40	0.51	0.70
113 + 208 AC/SC	0.65	0.70	0.76	0.49	0.60	0.74

**Fig. 4:**
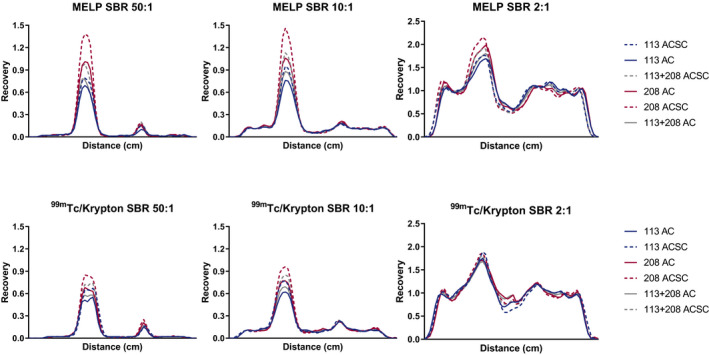
Line profiles on a voxel level across the 37 and 17 mm spheres. The medium energy low penetration line profiles are steeper, indicating a better spatial resolution.

Line profiles across the 37 and 17 mm spheres were drawn (Fig. 4) and present comparable differences between both collimators compared to the RC_mean_‐curves in terms of quantification. The line profiles derived from the MELP collimator are steeper, indicating a better spatial resolution for this collimator.

## 4 DISCUSSION

The goal of this study was to find the optimal combination of collimator (MELP vs ^99m^Tc‐Krypton), photopeak selection (113 vs 208 keV vs 113 + 208 keV), and scatter correction for ^177^Lu SPECT/CT image quality, quantification, and spatial resolution. Figure 2 shows that image quality using both the 113 + 208 keV photopeaks, is best with the highest CNR, especially in SBR 50:1 and 10:1. This observation can be explained by the fact that the combination of both photopeaks results in highest count statistics. However, in the clinically relevant SBR 2:1, the difference between solely the 208 keV and 113 + 208 keV is small: for example, CNR 10.3 for 208 keV AC and CNR 10.8 for 113 + 208 keV AC for the MELP collimator (Table 2). Furthermore, Fig. 2 shows that the MELP collimator meets this *Rose criterion* in smaller sphere sizes compared to the ^99m^Tc/Krypton collimator in SBR 2:1.

The highest RC_mean_ in this series was 0.9 for the MELP SBR 50:1 and 208‐keV AC/SC reconstruction in the 37‐mm sphere (Fig. 3). Overall, the recovery of the 208‐keV photopeak only was the highest and the addition of the 113‐keV photopeak decreases the recovery. For the MELP collimator, 208 keV AC/SC recovery was 0.84 for SBR 2:1 and 0.76 for 113 + 208 keV AC/SC. Recoveries of the ^99m^Tc‐Krypton collimator for these settings were 0.77 and 0.74, respectively. The additional noise that originates from the 113‐keV peak results in decreased quantitative accuracy. The lowest RC of the 113‐keV photopeak is caused by the lower counts statistics and increased noise around this photopeak compared to the other reconstructions. These facts result in lower recoveries, even though more septal penetration and scintillation could be expected.

In literature, mean recoveries of 1 have been observed for ^177^Lu in a large volume of 500 ml.^17^ Due to the limited spatial resolution and partial volume effects (PVE) of ^177^Lu gamma imaging, recoveries of 1 are hard to achieve in volumes below 100 ml. The degree of PVE is related to lesion size and since tumor sizes might change due to treatment effect, quantitative follow‐up measurements are challenging in small lesions. Post‐reconstruction corrections have been proposed to overcome PVE limitations.^17^ The proposed PVE correction method is feasible, yet again another step in the already extensive dosimetry workflow. The recent study of Peters et al. show ^177^Lu RC‐curves from the same SPECT/CT system as used in this study with spheres sizes up to 60 mm.^6^ The RC‐curves flatten with sphere sizes above 37 mm, therefore only lesions with diameter ≥ 37 mm should preferably be included in dosimetric analysis. RC‐curves are generally accepted tools in nuclear medicine to evaluate quantitative measurements with respect to lesion size, and provide insight in the degree of PVE for specific reconstructions.

The spatial resolution of the gamma camera is limited compared to other medical imaging techniques, and is in the order of 10 mm for ^177^Lu. The impact of PVE and spatial resolution are highly related, especially in smaller lesions, which decreases quantification reliability.^9^ Similarly to the RC_mean_ curves, the 208 keV AC/SC reconstruction resulted in the highest recovery. The recoveries observed in the line profiles were higher compared to the RC‐curves, as these were based on individual voxel values and the RC_mean_ was calculated using average activity concentrations. The MELP collimator line profiles are steeper compared to the ^99m^Tc/Krypton collimator, indicating better spatial resolution.

Overall, the MELP collimator showed a better performance in terms of image quality, quantification recovery and spatial resolution compared to the ^99m^Tc/Krypton collimator. This is probably due to the difference in septal thickness of 1.14 and 0.4 mm, respectively (Table 1). An increased number of redundant high energy photons are passing the septa of the ^99m^Tc/Krypton collimator, resulting in images with high noise levels and a lower CNR (Fig. 2).

A limitation of this study is that only spheres with homogenous radioactivity distributions were used for analysis, which is less representative of a clinical distribution within a tumor. Evaluation of other target geometries and heterogeneous activity concentrations would be interesting before selecting a protoco; however, this is not common practice. Such an approach is also not essential in the comparison between collimators, photopeak windows and scatter correction. In this study also, the number of iterations and subsets in the reconstruction protocol was not varied, which might have had some influence on the study outcomes. Yet, the effects of these reconstruction setting are far less than the choices for collimators, photopeak windows and scatter correction.

To conclude, based on the reported results in this study, clinical ^177^Lu SPECT/CT acquisitions in our institute are performed with the MELP collimator and the photopeak window is set at 208 keV with both attenuation and scatter correction to achieve the highest CNR, quantification recovery and spatial resolution. Based on this research, our other Symbia T SPECT/CT system was also equipped with a MELP collimator to enable flexibility in logistics and to have comparable image quality between both imaging systems. In addition, we decided to only include lesions > 20 mm for quantification and dosimetric analysis.

## CONFLICT OF INTEREST

The authors declare that they have no conflict of interest.
